# Because space matters: conceptual framework to help distinguish slum from non-slum urban areas

**DOI:** 10.1136/bmjgh-2018-001267

**Published:** 2019-04-11

**Authors:** Richard Lilford, Catherine Kyobutungi, Robert Ndugwa, Jo Sartori, Samuel I Watson, Richard Sliuzas, Monika Kuffer, Timothy Hofer, Joao Porto de Albuquerque, Alex Ezeh

**Affiliations:** 1 Warwick Medical School, University of Warwick Warwick Medical School, Coventry, UK; 2 African Population and Health Research Centre, Nairobi, Kenya; 3 Global Urban Observatory Unit, United Nations Human Settlements Programme, Nairobi, Kenya; 4 Faculty of Geo-Information Science and Earth Observation, Universiteit Twente, Enschede, The Netherlands; 5 Institute for Healthcare Policy and Innovation, University of Michigan, Ann Arbor, Michigan, USA; 6 Institute for Global Sustainable Development, University of Warwick, Coventry, UK; 7 Dornsife School of Public Health, Drexel University, Philadelphia, Pennsylvania, USA

**Keywords:** slums, urban, mapping, identification, definition

## Abstract

Despite an estimated one billion people around the world living in slums, most surveys of health and well-being do not distinguish between slum and non-slum urban residents. Identifying people who live in slums is important for research purposes and also to enable policymakers, programme managers, donors and non-governmental organisations to better target investments and services to areas of greatest deprivation. However, there is no consensus on what a slum is let alone how slums can be distinguished from non-slum urban precincts. Nor has attention been given to a more fine-grained classification of urban spaces that might go beyond a simple slum/non-slum dichotomy. The purpose of this paper is to provide a conceptual framework to help tackle the related issues of slum definition and classification of the urban landscape. We discuss:

- The concept of space as an epidemiological variable that results in ‘neighbourhood effects’.
- The problems of slum area definition when there is no ‘gold standard’.
- A long-list of variables from which a selection must be made in defining or classifying urban slum spaces.
- Methods to combine any set of identified variables in an operational slum area definition.
- Two basic approaches to spatial slum area definitions—top-down (starting with a predefined area which is then classified according to features present in that area) and bottom-up (defining the areal unit based on its features).
- Different requirements of a slum area definition according to its intended use.
- Implications for research and future development.

**Summary box**

- People who live in slums have worse health outcomes than those in formal city precincts; yet, slums are commonly not identified in censuses and hence in surveys which take their sampling frames from censuses.
- A large barrier to identifying slums lies in the lack of an agreed definition that can be applied on a routine basis. We describe the issues that must be confronted in the development of a standardised definition (or classification system) for slums.
- We show that the requirements of a definition/classification system vary according to the intended use of that definition/classification.
- We describe the implications of our analysis for research and for future developments in spatial epidemiology of cities.

## Introduction

Nearly a billion people live in slums according to UN-Habitat.^1^ People who live in slums are exposed to numerous hazards arising from poverty, poor services (transport, sewage, water and power), crime and dangerous locations (eg, flood plains). These factors are determinants of conditions such as gastrointestinal disease, malnutrition and poor mental health. Space is an important variable in epidemiology; ‘neighbourhood effects’ may result from variables that are correlated with geographic areas.^2^ Such neighbourhood effects are particularly likely to take place in densely inhabited slum areas where the physical environment is closely shared and where one person’s behaviour impinges on another’s.^3^ For example, lack of effective sanitation, poor nutrition, behavioural factors, crowding and other possibly unmeasured factors interact to generate the high rate of childhood death observed in slums.^3^ Space is therefore an important epidemiological variable net of individual risk factors such as poverty or level of education. Some have argued that the term ‘slum’ should be abandoned,^4^ but unless neighbourhood effects are disproven at some future date it will remain necessary to identify ‘spatial concentrations of poverty’, whatever we wish to call them. A recent Lancet series^3 5^ and Bellagio conference^6^ identified three purposes for identifying slum areas:

1. Scientific—in essence to study the putative neighbourhood effects on human outcomes as mentioned above.
2. For policy purposes, for example, to target investments and as the basis for advocacy.
3. To monitor expansion, contraction and upgrading of slums as per the Sustainable Development Goal 11 (target 11.1).

Whatever the reason, identifying slums requires that slum areas be distinguished from non-slum urban areas. Dictionary definitions, for example, ‘a squalid section of a city characterised by inferior living conditions and usually by overcrowding’,^7^ are vague and hence not suitable to distinguish slum from non-slum spaces for operational and scientific purposes. More specific definitions of slums have been put forward by organisations of the United Nations and by individual countries (table 1).

**Table 1:** Current definitions of slums

Source	Definition
UN-Habitat current definition—based on a household^23^	‘Any specific place, whether a whole city, or a neighbourhood, is a slum area if half or more of all households lack improved water, improved sanitation, sufficient living area, durable housing, secure tenure, or combinations thereof’.^20^ The criteria (improved water, etc) are defined in more detail.
UN original definition—based on an urban space^24^	‘A contiguous settlement where the inhabitants are characterised as having inadequate housing and basic services’.
India (2011 census)^25^	A compact area of at least 300 population or about 60–70 households of poorly built congested tenements, in unhygienic environment usually with inadequate infrastructure and lacking in proper sanitary and drinking water facilities.
Bangladesh (2014 slum census)^26^	A cluster of compact settlements of five or more households which generally grow very unsystematically and haphazardly in an unhealthy condition and atmosphere on government and private vacant land. Slums also exist on owner-based household premises.
Brazil (Brazilian Institute of Geography and Statistics definition)^27^	More than 50 contiguous households where most do not have their own property title of the land and live under one of the characteristics listed below: The absence of one or more services (energy supply, water supply, sewage system, garbage collection). Unplanned urbanisation.

It can be seen from table 1 that there is no agreement on how to define and hence identify a slum. In this paper, we do not attempt to derive such a definition. Rather, our purpose is to discuss the issues that must be confronted in the formation of any operational definition to distinguish slum from non-slum urban precincts. We also note that important information is likely to be lost in a slum versus non-slum dichotomy and we therefore consider the implications of our analysis for a more fine-grained classification of urban spaces. We start our analysis by discussing the ‘chicken and egg’ situation that the validity of a definition must be determined empirically but that such empirical enquiry requires a definition.

## An ontological or epistemological problem?

If slums could be identified by means of a specific reference standard based on underlying axioms or established scientific principles, then the ontological problem would have been solved and the empirical question would concern the consequences of living in a slum, just as a study could be mounted to determine the prognosis of a histologically confirmed disease. However, there is no such reference standard for a slum; this is the problem to be solved. One might suppose then, that a definition could be derived by studying the factors and combinations of factors that best portend the outcome(s) of interest. The medical analogy would be to base diagnosis on the combination of clinical features that provided optimal sensitivity and specificity. So, in the case of slums, the idea would be to work back from outcome (health and well-being) to determinant (slum vs non-slum). Such an exercise is beset by problems in the case of slum vs non-slum areas. These problems are logistical (the scale of the enterprise required), statistical (picking apart interactions between various determinants and outcomes)^2^ and methodological (cross-sectional studies are prone to strong selection and survivorship biases).^3^ Even if these problems could be overcome, a definitional problem would remain. First, outcomes are polychromous, meaning that a selection would have to be made regarding the outcome(s) of interest. Second, thresholds would have to be set for outcomes such as rates of mortality or disease to deal with inevitable trade-offs between sensitivity and specificity. To return to the medical analogy, the process of working back from outcome to a spatial definition is likely to be no more successful than the medical nosology before Virchow.^8^ Pending a possible solution to all the above problems, there is one remaining alternative: a consensus definition where some combination of indicators are defined as replicating the underlying construct of interest.^9^ In other words, unlike most entities to which standard psychometric theory is applied, we propose that there is no entity ‘slum’ that has an underlying reality which is reflected in the various factors by which we measure it. Rather, we propose that the measurement of slum is an operational one to be defined entirely by the measurement procedure.^10^ Such a composite model can then be iteratively refined through scientific studies to provide more accurate or parsimonious definitions or classification systems. Such was the case with respect to schizophrenia research, for example.^11^

We will now turn our attention to the issues that will have to be confronted or clarified in trying to distill a consensus definition. We start with the putative ‘building blocks’ for the slum concept.

## The building blocks: features that might contribute to a definition of slums

A large number of features have been proposed as characterising slums. These features can be classified in various ways. The method of Kohli *et al*,^12^ which focuses on what can be observed and measured from very high resolution satellite images, proposes an ‘ontology’ based on three levels: objects (eg, building materials of dwellings and lane layout), settlements (eg, population density) and environments (eg, gradient and surrounding of settlements). We have extended this somewhat and grouped typical examples of slum characteristics in table 2. This is a ‘long list’ of features from which anyone wishing to define a slum area may draw.

**Table 2:** Features that have been suggested as those that might help in characterising slums*

Built environment	Construction materials for houses especially floor, wall and roofing materials† Lay-out of lanes/buildings (haphazard vs organised; high vs low entropy); road width Density of living area (people per room or per square kilometre)†
**Services**	Water† Sanitation† Power (electricity (legal and illegal), gas) Schools Garbage removal (public/locally organised) Health facilities/services per unit of population Transport (Euclidean and Manhattan distances from work places and facilities)
**Ecology**	Gradient; altitude (floodplains, areas at risk of subsidence, landslides and other hazards) Green spaces Blue spaces Air quality Environment and industrial hazards
**Socioeconomic**	Security of tenure/title† Poverty level Access to amenities/place of work Stigma

*This list is not exhaustive, but covers many of the main features of slums found in the literature.

†Features included in the UN-Habitat definition (table 1).

Some of these features or ‘dimensions’ are not particularly specific to ‘slums’ (eg, situation on floodplain or air quality), while others are more specific (eg, poor sanitation, disorganised street layout and ‘shanty dwellings’). Some are much more easily quantifiable (eg, proportion of homes with no sewer connections) than others (eg, risk of subsidence). Notice that we have not included here features that are putative outcomes of living in a slum—crime, happiness, health, educational attainment, etc. This is because the purpose of defining or classifying urban spaces is to predict human health and welfare. We now turn our attention to the methods that can be used to identify and (to some extent) quantify the various features listed in table 2 that (may) define slums.

## Sources of data to identify and quantify features of slums

There are broadly three (non-exclusive) methods to collect data to inform characterisation and classification of spaces: household surveys, ground surveys of features identified in an area (rather than individual households) and Earth Observation imagery.

In table 3, we attempt to identify the strengths and weaknesses of various methods for identifying features of slums on the basis of the literature and our knowledge of the topic—we come later to the need for more research in this area. It is clear that different methods to identify features that might signify slums have their - individual strengths and weaknesses, and the extent to which one mechanism may be a proxy for another is uncertain. The use of Earth Observation to characterise spaces such as slums or distinguish them is evolving fast, and a recent review identified 87 studies describing the use of Earth Observation images for slum identification.^13^ However, some features may work well in one area but not in others.^14^

**Table 3:** Features of slums

Domain	Item	Household survey	Ground survey for features of an area	Earth observation	Comment
Built environment	Durability of construction materials	++++	+++	++	Spectral analysis can be used to get some idea of roof materials (especially with ultra-high resolution)
	Layout of lanes and orientation of structures—degree of entropy	++	+++	++++	Earth observation images can be used to quantify this characteristic, for example, using advanced image feature extraction and classification methods such as machine learning
	Density, for example, people sleeping in same room/people per square km	++++	+	+	Clearly, this must be a proxy measurement unless based on household survey
Services	Water	++++	+++ (hard to quantify)	−	
	Sanitation	++++	+++	+	Open sewers discernible on very-high-resolution images
	Power	++++	+++	+	Use of night-time light images allow to detect availability of street lighting but the resolution is limited^25^
	Solid waste management	+++	+++	++++	
	Health and education facilities	++++	+++	−	
Ecology	Flood plain	−	++	++++	
	Probability of subsidence	−	++	++++	Amount of subsidence can be measured accurately from space with radar-based interferometry
	Green and blue space	+	++	++++	
Socioeconomic (social exclusion)	Security of tenure/title	+++	+	−	
	Level of poverty	++++	++	(++)	The extent to which earth observation images may be a proxy is unknown^28^
	Crime and safety	++++	−	−	
	Social capital	++++	+	−	

But identifying and selecting features to be used in defining a slum is only the first step. Next, these features need to be combined in some way.

## Two basic approaches to combine features to define a spatial concept

From a practical perspective, there are two basic mechanisms for classification of a space on the earth’s surface, such as slum versus non-slum (or to various subtypes).

1. *Features first (bottom-up) method*. Here the area to be classified as slum (vs non-slum) is built up from observed features (eg, a certain number of contiguous dwellings have certain features in common). The essential point is that the features-first method does not start with a predefined spatial unit, but with a survey. Spatial boundaries are then fitted according to what is observed. This is the method used in the country and UN original definitions in table 1.
2. *Space first (top-down) method*. Here an area is demarcated and is *then* classified as slum versus non-slum. The UN-Habitat definition (table 1) follows this approach. This area could be a piece of land surrounded by natural or ‘man-made’ boundaries—a triangle with a river on one side and roads on the other two sides, for example. In many cases, such an area will already have a label—for example, famous slums like Kibera (Nairobi), Dharavi (Mumbai) and Makoko (Lagos). Many important surveys, such as Demographic Health Surveys, build their sampling frames from censuses, so the use of census enumeration areas as spatial units holds promise. However, surveys that are based on censuses are obliged to follow an algorithm that randomly ‘displaces’ households by up to 2 km (in urban areas) in any direction in order to protect anonymity. This means that, in order for a person in a survey to be identified as slum resident, it is necessary for two things to happen. First, the census tract must be labelled as slum or non-slum. Second, the person or household must be identified as originating in a slum or non-slum precinct so that this can be picked up in a survey.

## Quantifying and combining features to define slums

Assuming, for the time being, that a slum is not to be identified on the basis of a single feature (such as population density or degree of entropy), then the different features must be combined in some way, and thresholds determined, such that the combination of features yields a slum classification system.

### Aggregating household data to yield a quantitative measure

Here, data from household surveys are aggregated at an area level. Since such data are collected in censuses, aggregating these data to the level of census enumeration areas would be highly cost-effective. Each enumeration area could then be classified as ‘slum’ or ‘non-slum urban’. Typically, an enumeration area in a slum would contain about 100 households. By a considerable margin, the simplest method would be to set a threshold for the proportion of households in a census tract that met the UN-Habitat criteria. For example, when >50% of households ‘qualify’, then this is a slum tract, as per the UN-Habitat World Cities report cited in table 1. This method lends itself to a more multilayer typology by simply specifying more than one threshold. The alternatives are either informed judgement or an algorithm for the combination of features but further work in an urban context is required (see below). However, algorithmic methods of aggregation are complex to the degree that agreement over which method to use would be very difficult to achieve for a simple slum vs non-slum dichotomy let alone a more fine-grained classification system. In the online appendix, we describe two interchangeable methods (a sequential algorithm and a scoring system) to illustrate this problem.

10.1136/bmjgh-2018-001267.supp1 Supplementary data

### Area-wide observations

All but the UN-Habitat method in table 1 are based on features identified from area surveys, rather than some sort of amalgamation of household features. It can be seen from table 1 that the methods used to date have been largely subjective, based on qualitative criteria (such as ‘most’, ‘usually’ and ‘generally’) and, as a result, the various features are not suitable for algorithmic agglomeration. Accepted uses of earth observation imagery include identifying changes in land use between censuses, ensuring censuses or surveys do not omit population clusters, and making observations in places (such as conflict areas) where censuses are not conducted. It is perhaps tempting to surmise that improvement in imaging will help solve the problem of distinguishing slum from non-slum areas or in deriving a finer-grained classification. However, imagery cannot pick up ‘social features’ such as home ownership and ‘machine learning’ is hampered by the lack of a reference standard—the ‘chicken and egg’ situation referred to above.

## Requirements of a method to define slums according to the use to which the definition will be put

To make a common and reliable consensual definition (or classification system) it would be necessary to agree:

1. Which features (from table 2) should be included?
2. How they should be observed?
3. How they should be dichotomised (or quantified)?
4. What weight they should have?
5. Whether or when to use a bottom-up or top-down approach?
6. How the selected features should be combined, taking account of interactions?

Both the degree to which the features are essential to the definition of a slum (in terms of defining the concept with its hypothesised theoretical relationship to health and well-being), and the reliability with which each feature can be measured individually must be considered. Unlike the more familiar approach to psychometric measurement, the features included by definition in a composite index must be both comprehensive and finite. Socioeconomic status is one of the most familiar examples and based on Weber’s views about the dimensions of social class is captured by income, education and occupational status.^15^ All three are required and the addition of any other feature would change the concept.^16^ Hence, the challenge of constructing a composite is establishing a method by which candidate features will be selected for inclusion, likely employing some sort of consensus process.

While harmonisation of definitions across countries is ultimately required if there is to be long-term conceptual coherence, we can imagine one use where harmonisation is unnecessary, one where it is desirable but by no means essential and one where it is essential:

1. *For local policy/management and advocacy*. Here a country may determine its own definition to identify slum areas as India, Brazil and Bangladesh have done. If the purpose is simply to identify slums so that growth or contraction of slums could be monitored within country, then all that is required is that the method is consistent over time and has some local content validity as representing the concept of a slum and it proves useful in a locally defined way. Bird and colleagues provide an excellent account of what is possible if slums are identified in censuses, tracking how both health and the determinants of health have improved over two census epochs.^17^
2. *For scientific explorations of spatial determinants of health and well-being and for evaluation of interventions*. Here, while a common definition would be ideal, some variability would not invalidate scientific study but samples would need to be sufficiently large to compensate for the variability introduced by the definitional differences. Sensitivity would be more important than specificity since definitions could be tightened up iteratively on the basis of successive studies (see below).
3. *To compare the extent of slums across countries and to measure international progress in reducing slums*. Here, the important requirement for comparisons would be a common standard and consistent definition. If definitions differed or there was inconsistency in the application of a given definition, then the results would be misleading as definitional differences would not be distinguishable from differences in progress across country^18^; for example, in the case of the Bangladeshi and Brazilian definitions in table 1.

## Implications for further enquiry

Three types of correlation are relevant to our quest.

### Correlations between features in a slum

These correlations can be studied between single features or across combinations of features as high levels of correlations suggest potential redundancy in the features used to define a slum. An example of the former is the extent to which entropy is a proxy for population density. Studies of combinations of features could explore, by way of example, the extent to which features observed on geospatial images are proxies for UN-Habitat features. If a reference standard could be agreed, for example, based on the Brazilian definition, then the accuracy (sensitivity and specificity) of more parsimonious combinations of features could be determined. The data collected by countries that are attempting to implement identification of slums in their censuses will help with the above questions.

### Correlations between areas currently called slums and various features that make up slums

It has been said of slums that, like family resemblances, people ‘know it when they see it’. This notion embodies the idea that some things are identified tacitly. Two questions follow from this line of thinking. First, what is the interobserver variation when many people look at the same place? Second, insofar as there is agreement, what is driving agreement? The first question is easier to answer than the second, since the degree of agreement can be measured in standard ways. However, working out how people are weighting and combining the different features to reach consensus or the lack of it would be tricky. It is likely that while some places elicit a uniform response (slum or non-slum) many others split the vote. Nevertheless, given high interobserver agreement (say, kappa >0.6) then a machine learning classifier could be trained to recognise slums and distinguish them from non-slums. In this way, we may progress iteratively through intuitive ideas of slum to more highly specified definitions, then through semiautomated methods and ultimately fully automated definitions.^19^

### Research into how slum features correlate with human outcomes

As stated in connection with candidate features of slums, a conceptual distinction is required between the determinants (proximal causes) of impeded human health and welfare and human health and welfare itself. Slum identification may be an efficient way to identify populations subject to particularly important threats to human welfare, with the ultimate goal of intervening to prevent those threats from materialising. Together and in combination, these features constitute the independent or explanatory variables in studies where the dependent or outcome variables relate to health and welfare. Definitions could be refined as more information was collected bearing in mind the importance of longitudinal studies wherever possible.^3^ Given enough information, one given area could be graded into more than two risk categories.

## Future trends: beyond a slum versus non-slum dichotomy?

The world is changing rapidly and satellite images are but one type of data that can be collected on a routine basis. Data obtained from mobile phones and the ‘internet of things’ can be combined with participatory community data and earth observation to provide ever richer information to inform policy and identify areas that are at high or increasing risk.^20 21^ As methods evolve, finer-grained classifications should be possible covering slum areas of different severity categories and identifying small cities and periurban areas where risks to health and welfare should be better understood. Returning to a theme in the introduction, while there are good arguments to identify spatial constructs where various factors interact to produce neighbourhood effects, there are also good reasons to identify and attend to specific risks, irrespective of where those risks apply. Thus, data collected in order to identify areas where multiple risks interact, are also applicable to identification of areas according to specific risk factors. The intensity of these risks can be visualised as ‘heat maps’^20^ and other visualisation methods, which can facilitate reflexive policy responses for conditions such as malaria.^22^ However, while tracking specific causes of specific events will undoubtably prove useful, it is important not to loose sight of neighbourhood effects resulting from complex interactions and variables not observed and hence not included in the risk prediction model. These neighbourhood effects related to health and development outcomes should be studied across slum and non-slum areas or, better still, across urban areas classified into more than just two categories.

## Conclusion

Identifying and analysing the geographic clusters in which people are located is recognised as a productive way to learn about population health. People living in a slum area share many geographically determined microbiological, physical and social risks and hence one expects these neighbourhood effects to be strong. These environmental determinants of disease have been recognised for at least four decades. But the process of formulating questions, applying for funding, collecting data, analysing data, assimilating data and acting on new knowledge has been cumbersome. New tools are becoming available to make this whole process more dynamic with earth observation instruments and new methods for collecting and analysing data on the ground in real time. As enquiry and action become more closely coupled, the distinction between research and management is becoming eroded. In order to capitalise on the new opportunities, it will be necessary to work out how the determinants of disease can be represented in space in order to curtail or forestall the diseases themselves. We offer this paper as a step on this journey with respect to circumstances where space itself is an epidemiological variable, not just the surface onto which epidemiological data are projected.
