# Supplementary material for: Because space matters: conceptual framework to help distinguish slum from non-slum urban areas
Source: BMJ Glob Health. 2019 Apr 11;4(2):e001267. doi: 10.1136/bmjgh-2018-001267 (PMC6509608; doi:10.1136/bmjgh-2018-001267)
Supplement: Supplementary data [file bmjgh-2018-001267supp001.pdf]

APPENDIX: EXAMPLES OF TWO METHODS TO DEFINE A SPATIAL CONSTRUCT BY AGGREGATING HOUSEHOLD CHARACTERISTICS

1. Sequential algorithm. The aggregated results of household surveys are translated into a definition by means of an algorithm such as that in Figure A1 (provided purely as a specimen).

Figure A1. Specimen algorithm to define slums – this is an example and there is no consensus as to how a slum can be defined at this level of specificity.

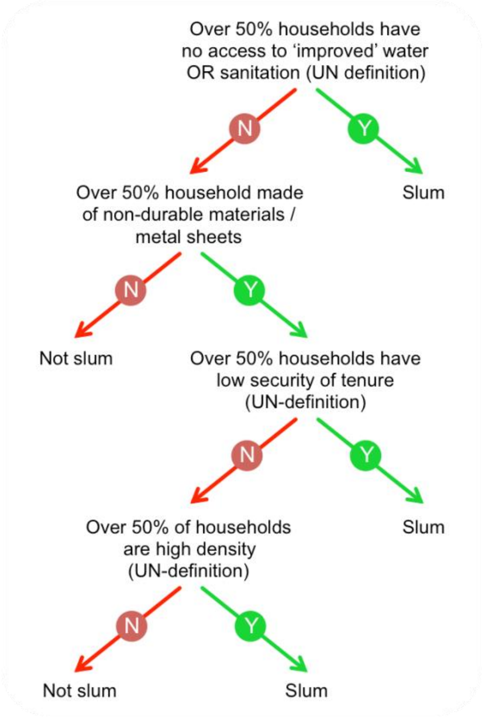

The problem with such an algorithm is that it is driven by the starting criterion and sequence (in the above case of water and sanitation) and subsequent sequencing features. For this reason, it would likely be difficult to secure a widely agreed method.

2. A scoring system. This represents a 'weighting' system rather than the 'gating' system explicated under method 1. An example is given in Table A1. In this particular example, the products of the proportions of households that meet each UN-Habitat criteria and their

‘weight’ would be summed and some threshold applied to designate a space as slum or non-slum.

Table A1: A scoring system for slum classification

**Feature***	**Weight****
Lack of ‘improved’ water	10
Lack of ‘improved’ sanitation	10
Crowding	8
Security of tenure	7
Non-durable buildings	5

**Each defined by UN-Habitat criteria (thresholds) in this example, but in practice a cut-off for each criterion would be required.*

***For illustration purposes only*

Designation of slum could be determined by the following formula: $\sum_{5}^{1}(p \times w) > x$*, where p is the proportion meeting a certain criterion and w is the weight for that criterion. For example, if 50% of households met the UN-Habitat criteria for each feature the score would be 5+5+4+3.5+2.5=20*

The difficulty with both of the above methods lies not only in deciding the cut-off value for each feature, the relative importance of each criterion, and which criteria to include, but also in taking into account their interactions or potential trade-offs. In the absence of a clear scientific rationale for any particular method, it would be difficult to reach an agreed method for widespread application.
